# Sodium valproate and gabapentin reduce the seizure-like behavior induced by pentylenetetrazol and mechanical stress in *Drosophila melanogaster*: sex influence on behavioral responses

**DOI:** 10.1007/s00702-026-03140-0

**Published:** 2026-04-02

**Authors:** Gabriele dos Santos, Adeline Alice Dalbem Goes, Mauro Schneider Oliveira, Getulio Nicola Bressan, Roselei Fachinetto

**Affiliations:** Programa de Pós-Graduação em Ciências Biológicas: Farmacologia, Centro de Ciências da Saúde, Universidade Federal de Santa Maria, Santa Maria, RS 97105-900 Brazil

**Keywords:** Epilepsy, *Drosophila melanogaster*, PTZ, Anticonvulsants, Sex differences, Experimental model

## Abstract

Epilepsy is a multifactorial neurological condition characterized by abnormal, recurrent neuronal discharges. The search for new anticonvulsant treatments requires experimental models that replicate the pharmacological responses of drugs commonly used for this condition. This study aimed to evaluate the effect of pentylenetetrazol (PTZ), a pro-convulsant agent, combined with thermal or mechanical stress, on seizure-like behavior in female and male *D. melanogaster*. Moreover, to determine the response to anticonvulsant drugs. Initially, the locomotor activity of the flies was evaluated after exposure to PTZ (0.1–60 mM) to rule out potential baseline locomotor deficits that could interfere with the results. Next, the flies were exposed to PTZ for 24 h and subjected to thermal or mechanical stress to induce seizure-like behavior. From the results, exposure to PTZ (1 mM) associated with vortex-induced mechanical stimulation was selected for inducing seizure-like behaviors in both male and female flies. Subsequently, the groups were treated with anticonvulsant drugs, sodium valproate (0.1, 1.0, 10 mM) or gabapentin (0.5, 1.0, 2.5 mM). Both drugs attenuated the seizure-like behaviors induced by PTZ and mechanical stress, showing higher effects in males. In conclusion, PTZ, associated with mechanical stress, reproduces a phenotype resembling seizure-like behavior in male and female *D. melanogaster*. Furthermore, the model was responsive to sodium valproate and gabapentin, especially in male flies, supporting its translational potential for identifying new drugs and reinforcing the importance of including sex as an experimental variable.

**Graphical abstract:**

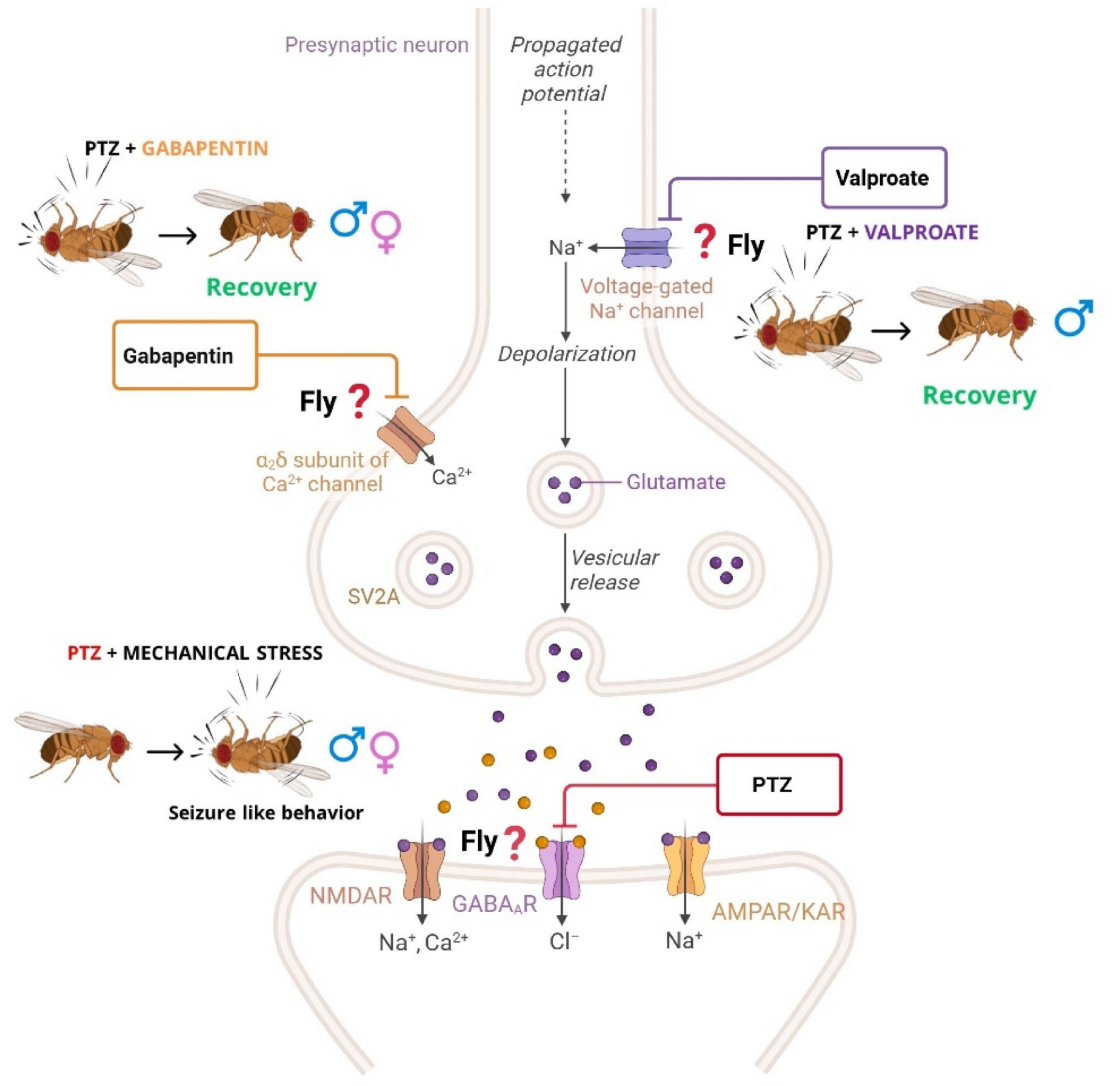

**Supplementary Information:**

The online version contains supplementary material available at 10.1007/s00702-026-03140-0.

## Introduction

Epilepsy is a complex, multifactorial neurological disorder characterized by recurrent seizures resulting from abnormal, synchronous, and excessive neuronal discharges in the central nervous system (Fisher et al. 2005). These seizures may arise from genetic, structural, metabolic, immunological, or infectious factors and are often accompanied by cognitive, behavioral, and social impairments that profoundly affect the quality of life of affected patients (Scheffer et al. 2017). According to the World Health Organization (WHO 2019), approximately 50 million people worldwide are affected by epilepsy, with around 15 million experiencing forms that are refractory to current pharmacological treatments, highlighting the need for novel therapeutic approaches.

Beyond the neurological symptoms, epilepsy has significant effects on cognitive functions, including memory, language, and learning, particularly when brain regions such as the hippocampus and temporal lobe are affected (Howard and Baraban 2017). From a psychological perspective, individuals with epilepsy often exhibit elevated rates of depression, anxiety, and social exclusion, which can substantially hinder their integration into academic and professional environments (Singh and Goel 2021). Therefore, understanding the underlying pathophysiological mechanisms and developing novel therapeutic strategies are essential steps toward achieving more effective management of epilepsy*.*

Experimental models, particularly in rodents, have been widely employed due to their capacity to replicate central behavioral and neurochemical aspects of seizure-like activity (Löscher 2017). However, the initial screening of compounds with possible anticonvulsant effects often requires the use of many animals to determine dose–response and sex-specific effects. Consequently, the pharmacological identification of promising anticonvulsant molecules needs simpler and faster models for preliminary drug selection before testing in rodents. In this context, *Drosophila melanogaster* could be an alternative, combining a high reproductive rate, ease of maintenance, short life cycle, and a well-characterized genome with the conservation of genes encoding proteins involved in neurotransmission pathways (Reiter et al. 2001; Parker et al. 2011).

In rodent models, seizure-like behavior can be induced by different stimuli, including chemical, mechanical, and electrical approaches, as well as through genetic manipulations, to investigate the underlying mechanisms and potential treatments for epilepsy (Wang et al. 2022). Pentylenetetrazol (PTZ) is a chemical pro-convulsant agent with high bioavailability in the central nervous system of rodents (Monteiro et al. 2024). PTZ elicits a wide range of dose-dependent seizure types, those seen in humans (Akyuz et al. 2021; Monteiro et al. 2024), enabling the study of neuronal excitability and the evaluation of various molecules with anticonvulsant effects (Löscher 2017; Monteiro et al. 2024).

Although the PTZ model is well established in mammals, its application in alternative models, such as *Drosophila melanogaster*, remains in the early stages (Fischer et al. 2024). This gap represents a promising avenue for advancing methodological approaches in preclinical epilepsy research, particularly those that enable greater scalability, improved cost-effectiveness, and reduced use of vertebrate animal models.

Furthermore, one factor that remains poorly explored in preclinical rodent models of seizure-like behavior is the possible differences between females and males. Most seizure studies in rodents have been conducted primarily in males, thereby overlooking hormonal influences and potential sex-related differences in pathophysiology and therapeutic responses (Zucker and Prendergast 2020; Shansky and Murphy 2021). These can compromise the translation of findings to females, maintaining a bias in this area of biomedical research.

Given this context, the present study aims to validate an experimental model of seizure-like behavior in *Drosophila melanogaster*, using PTZ as the inducing agent and assessing mechanical and thermal stimuli as facilitators of the phenotype. Two drugs clinically used as anticonvulsants, sodium valproate and gabapentin, were employed to evaluate the model's pharmacological responsiveness. Furthermore, all experiments were conducted in both females and males to compare behavioral and pharmacological effects. The inclusion of sex as a variable from the early stages of research also aims to promote a more inclusive and representative approach in scientific investigation.

## Methodology

### Drosophila melanogaster

*Drosophila melanogaster* wild-type (Oregon-R strain), 1–4 days post-emergence, were maintained in an incubator at 25 ± 1 ºC, 60% humidity, and a 12-h light/dark cycle. The flies were reared in glass bottles containing a medium composed of glucose syrup (7.60%), corn flour (7.30%), soy flour (0.90%), agar (0.58%), yeast (1.73%), and Nipagin® (0.07%). For exposure to PTZ or anticonvulsant drugs, a treatment medium consisted of agar (1%), sucrose (2%), dry milk (1%), yeast (1%), and Nipagin® (0.07%). All drugs were commercially sourced from Sigma-Aldrich® (São Paulo, Brazil) or other certified suppliers to ensure purity and quality.

Behavioral tests were performed using adult flies aged 1 to 4 days post-emergence. Flies were lightly anesthetized on ice for 30 s, separated by sex, and then subjected to treatment with PTZ and anticonvulsant drugs. The number of independent experiments is depicted in Supplementary Material 1.

## Experimental design and treatment

### Step 1—exposure to pentylenetetrazol (PTZ) combined with thermal or mechanical stress

**Fig. 1:**
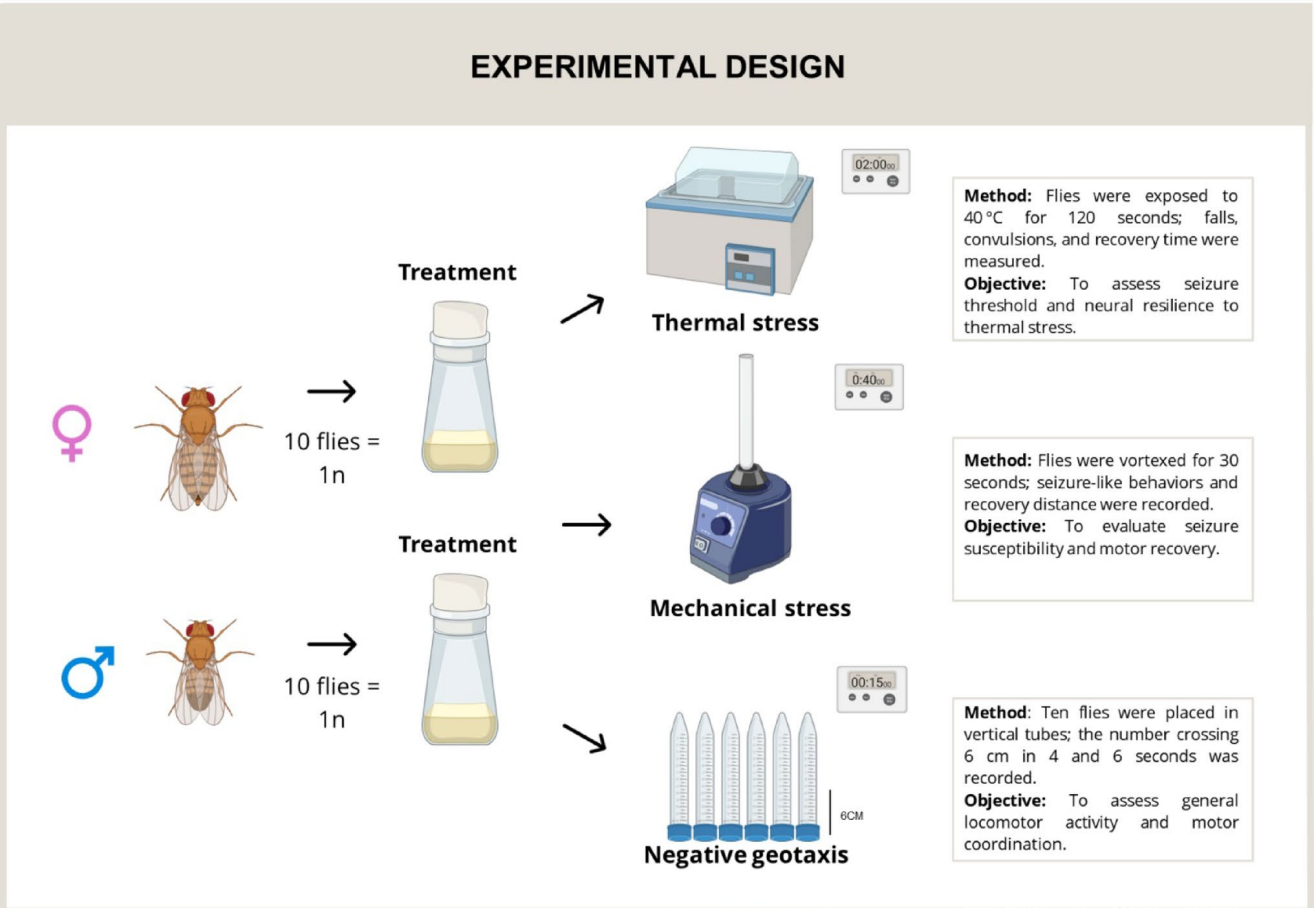
Experimental design. In Step 1, female and male *D. melanogaster* were exposed to PTZ (0.1–60 mM) for 24 h and subjected to thermal or mechanical stress to assess seizure-like behavior. In Stage 2, flies treated with PTZ (1 mM) were co-treated with gabapentin or sodium valproate and subsequently subjected to the mechanical resistance test. Images were generated using BioRender (Created in https://biorender.com/44xdmgc)

Flies were exposed to different concentrations of PTZ (0–60 mM) for 24 h. Subsequently, they were subjected to thermal (Bressan et al. 2023) or mechanical stress (Mituzaite et al. 2021) to investigate the influence of stress type on PTZ-induced effects, considering seizure-like behavior and recovery (Fig. 1). Seizure-like behavior was evaluated in both female and male *D. melanogaster* to examine sex-specific responses and identify potential differences in the phenotype. Locomotor activity was also assessed to determine possible effects of PTZ, which could influence seizure-like manifestations.

### Step 2—Effects of anticonvulsant drugs, sodium valproate and gabapentin, on seizure-like behavior induced by PTZ and mechanical stress

Following the initial behavioral screening, the mechanical stress assay combined with PTZ treatment was selected because it reproduced seizure-like behavior. Then, this method was used for subsequent experiments involving the anticonvulsant drugs sodium valproate and gabapentin. This selection was based on its better reproducibility, sensitivity, and manifestation of the seizure-like phenotype compared to the thermal stress assay. The effects of PTZ or mechanical stress were tested alone; however, no seizure-like behavior was visualized (data not shown). The mechanical stress added after PTZ treatment allowed the identification of seizure-like effects of PTZ and was therefore employed during the drug response evaluation phase. PTZ at 1 mM was chosen because it reproduced the seizure-like phenotype in female and male flies. From this stage onward, flies were treated with 1 mM PTZ for 24 h in combination with clinically used anticonvulsants (sodium valproate and gabapentin), followed by mechanical stress. The concentrations of drugs were determined based on previous literature data and re-tested in the present study using dose–response curves (Fig. 1). Sodium valproate was tested in the concentrations of 0.1, 1, and 10 mM (Yi et al. 2013), while gabapentin was tested at concentrations of 0.5, 1, 2.5, and 5 mM (Jang et al. 2023). These drugs were selected because of their distinct mechanisms of action: sodium valproate increases GABA levels and modulates sodium and calcium channels (Romoli et al. 2019) while gabapentin acts on the α2δ subunit of calcium channels, modulating neurotransmitter release (Hakami 2021a). The diversity of mechanisms allows a better understanding of the model's responsiveness to different classes of anticonvulsant drugs.

### Behavioral tests

#### Negative geotaxis test

To assess locomotor activity, 10 flies from each group were placed in transparent Falcon tubes (95 mm × 27 mm) marked with a 6 cm line. The tubes were positioned vertically under a fluorescent lamp approximately 20 cm above, and the flies were gently tapped to the bottom (Sudati et al. 2013; Figueira et al. 2017; Bressan et al. 2023). The number of flies crossing the 6 cm line within 4 or 6 s was recorded. This test evaluates locomotor activity, serving as a control to ensure that observed effects in seizure-like tests are not attributable to general motor deficits.

#### Thermal stress test

**Fig. 2:**
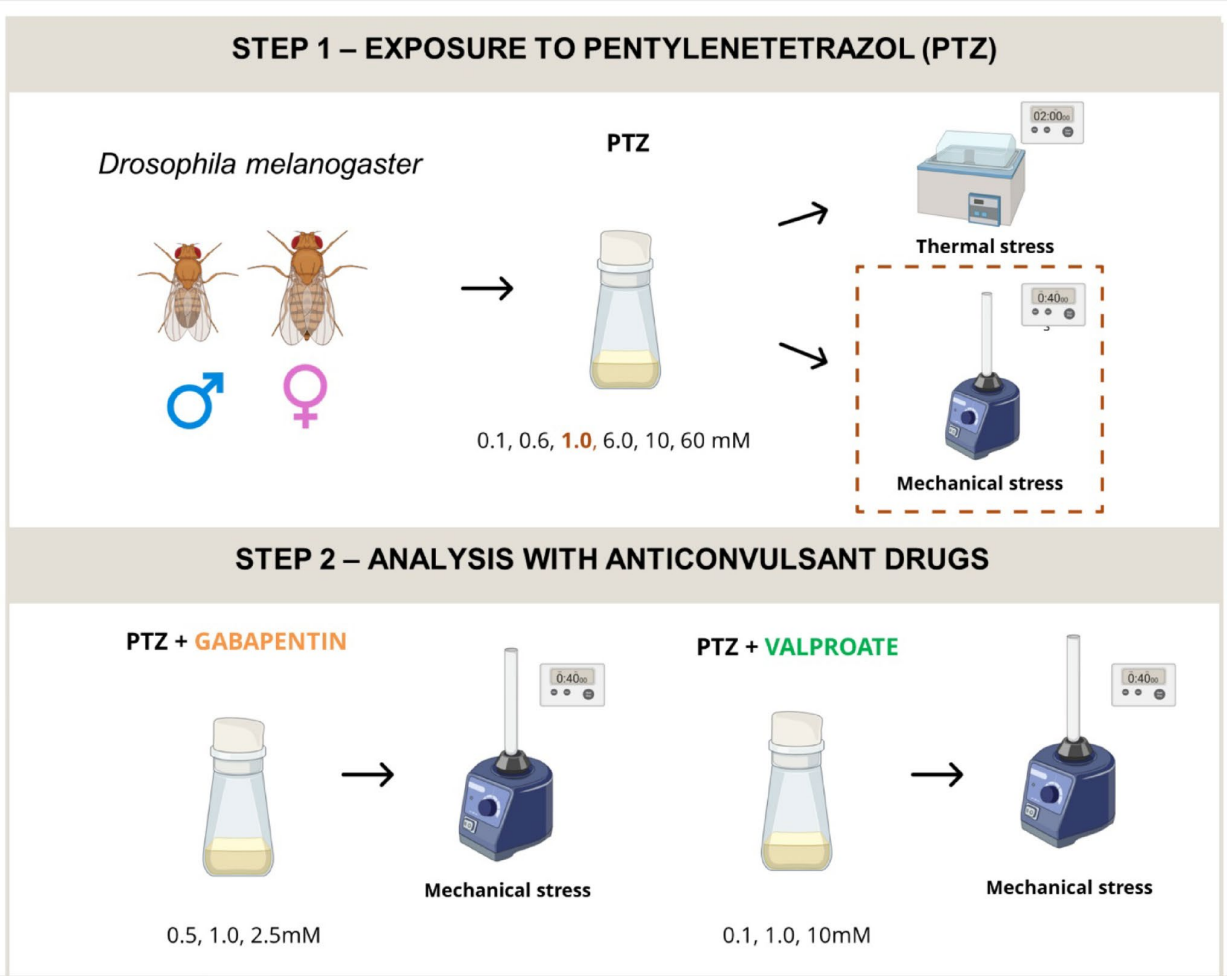
Summary of the experimental behavioral assays used to evaluate seizure-like phenotypes and motor performance in *Drosophila melanogaster.* Images were generated using BioRender (Created in https://biorender.com/44xdmgc)

The flies were exposed to different concentrations of PTZ (0—60 mM) for 24 h. To separate the female and male flies, they were anesthetized on ice for 30 s. Next, they were subjected to a thermal stress test, submerging the tubes in a water bath at 40–41 °C for 2 min, maintaining a constant temperature throughout the experiment (Neely et al. 2011; Mituzaite et al. 2021; Bressan et al. 2023). The number of falls (loss of posture) and uncoordinated and random movement of the wings were counted every 20 s (Fig. 2). After this period, the tubes were removed from the water bath, and the time for recovery of all flies was recorded.

#### Mechanical stress test

**Fig. 3:**
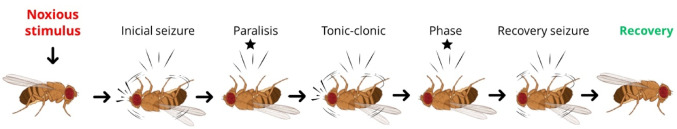
Seizure-like behavior in *Drosophila melanogaster*. Adapted from (Parker et al. 2011) (Parker et al. ). Images were generated using BioRender (Created in https://biorender.com/44xdmgc)

Vials containing 10 flies per treatment were subjected to vigorous agitation using a vortex for 30 s (Fig. 2). Following this procedure, seizure-like behaviors, including irregular wing flapping, loss of posture, and circular movements, were observed and quantified as previously described (Mituzaite et al. 2021). The observed behavior was classified according to the phenotypic description of tonic–clonic-like seizures in *Drosophila* (Parker et al. 2011) which includes periods of inactivity, motor discharge, and recovery with a refractory interval (Fig. 3). Given that the ability to recover rapidly after a seizure-like episode is critical, we standardized a new method to assess locomotor recovery in flies. This method consists of evaluating the number of flies capable of climbing 3, 6, 9, and 12 cm of a glass tube within 3, 5, and 10 s. Additionally, the number of flies remaining at the bottom after 10 s was recorded and classified as non-recovered.

### Statistical analysis

The data were analyzed using one-way or two-way ANOVA, followed by Tukey’s post hoc multiple comparisons test when appropriate. The significance threshold was set at * p* < 0.05. Results are presented as mean ± standard error of the mean (SEM). To evaluate potential sex differences or interactions between sex and treatment, data were analyzed separately for females and males, with “sex” included as an independent factor in the two-way analyses. To evaluate possible differences between the experimental groups, one-way ANOVA was used.

## Results

### PTZ alters seizure-like behavior in *D. melanogaster* submitted to mechanical stress

**Fig. 4:**
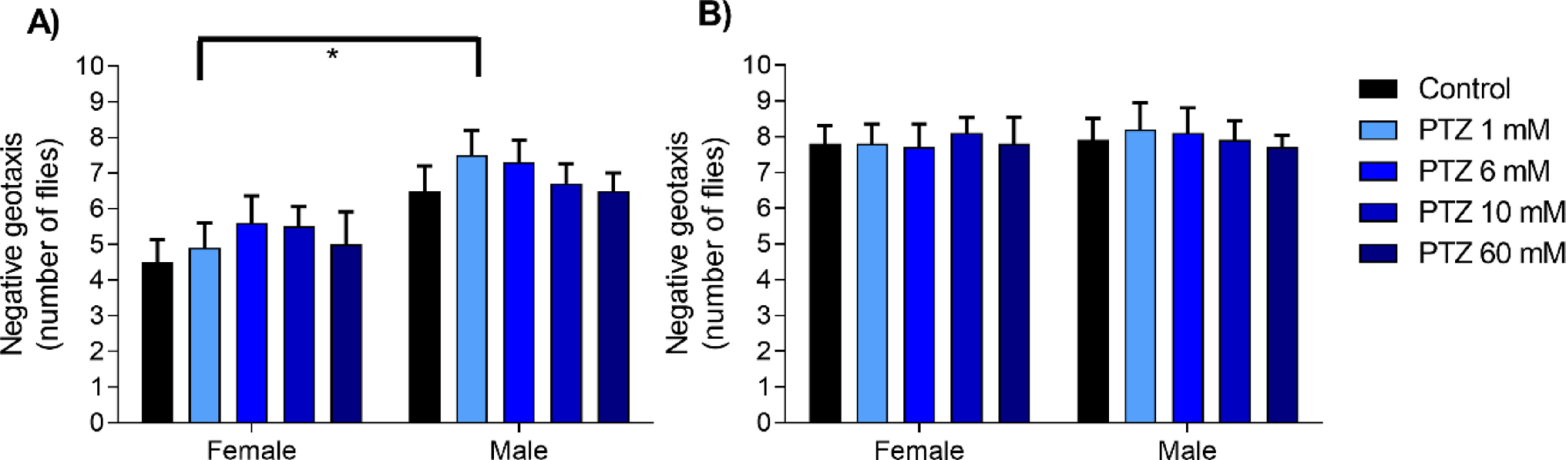
Effect of 24-h PTZ exposure on the locomotor activity of *Drosophila melanogaster*. **A** Locomotor performance at **A** 4 s and **B** 6 s in females (*n* = 10) and males (* n* = 10), assessed using the Negative Geotaxis Test. Data are presented as mean ± SEM. Statistical analysis was conducted using one-way and two-way ANOVA followed by Tukey’s post hoc test. *P* < 0.05 indicates significant differences between females and males under PTZ 1 mM treatment

Locomotor behavior of flies exposed to PTZ for 24 h was evaluated through the negative geotaxis test. PTZ (0–60 mM) per se did not alter the locomotion of flies, neither when quantified after 4 s (Fig. 4A) nor 6 s (Fig. 4B). A statistically significant difference was observed between sexes at 4 s (F (1, 45) = 19.78 and *p* < 0.0001, Fig. 4A) without effect at 6 s.

**Fig. 5:**
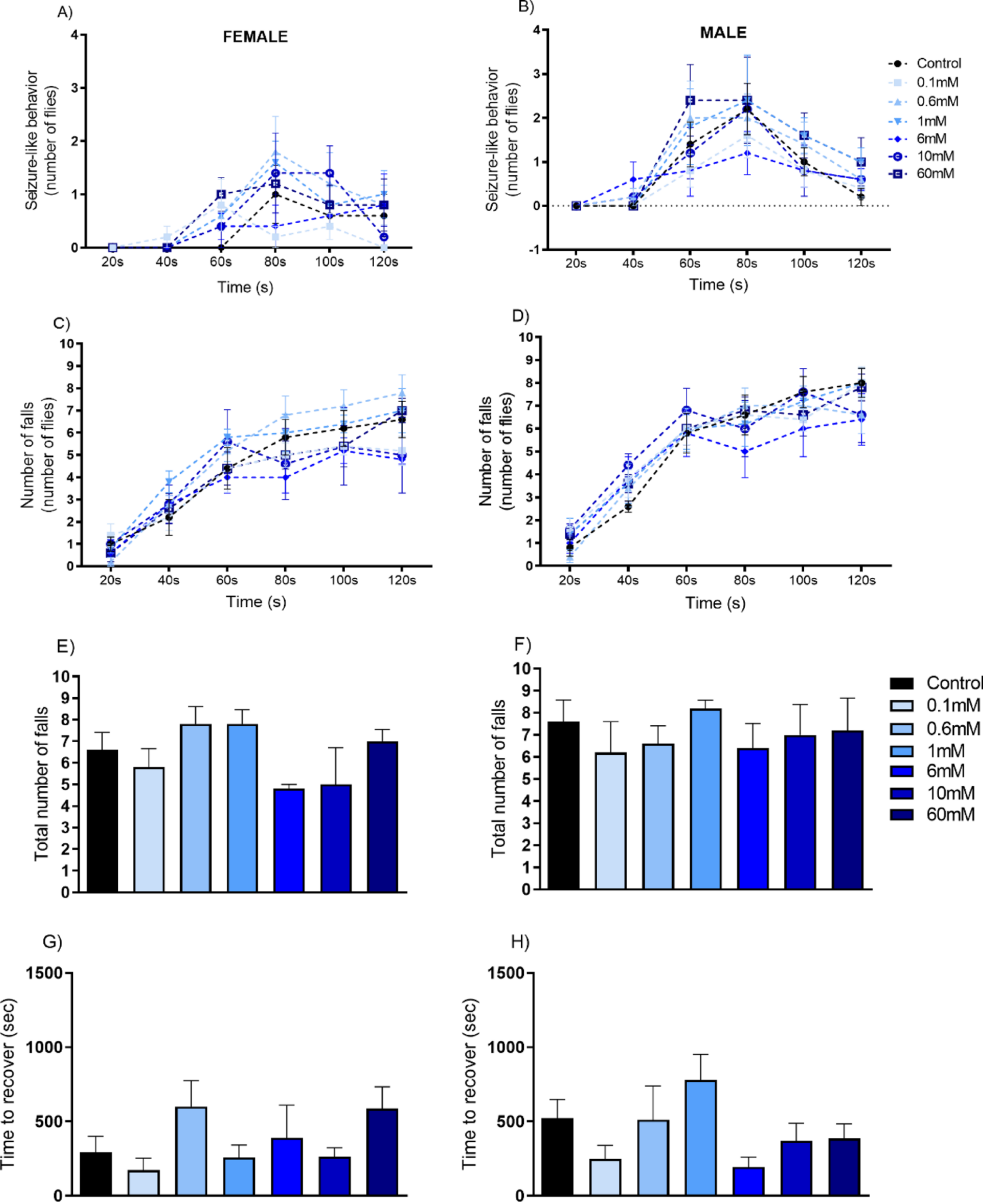
Effect of 24-h PTZ exposure on *Drosophila melanogaster* followed by thermal stress. **A**, **B** Number of flies presenting seizure-like behaviors was recorded every 20 s in females and males, respectively. **C**, **D** Number of flies that fell to the bottom of the vial was recorded every 20 s in females and males, respectively. **E**, **F** Total number of flies that fall during 2 min at each PTZ concentration in females and males. **G**, **H** Recovery time after thermal stress in females and males. Data are presented as mean ± SEM. Statistical analysis was performed using two-way ANOVA followed by Tukey’s post hoc test to compare sexes, and one-way ANOVA to assess the effects of PTZ concentrations within groups

Figure 5 shows the manifestation of seizure-like behavior evaluated during 2 min after treatment with PTZ for 24 h and subsequent exposure to thermal stress in female and male flies. No significant differences were observed in the number of flies presenting seizure-like behaviors recorded every 20 s (Fig. 5A—females and 5B—males), number of flies that fall to the bottom of the vial recorded every 20 s (Fig. 5C—females and 5D—males), total number of flies fall for 2 min (Fig. 5E—females and 5F- males) and time to recover after the thermal stress (Figs. 5G—females and 5H—males).

**Fig. 6:**
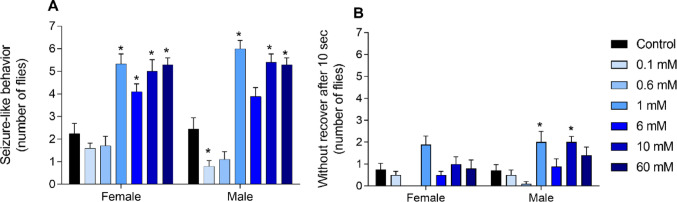
Effect of PTZ exposure on *Drosophila melanogaster* subjected to mechanical stress. **A** Number of flies exhibiting seizure-like behaviors. **B** Number of flies that failed to recover (remained at the bottom of the vial) after 10 s. Data is presented as mean ± SEM. Statistical analysis was conducted using one-way and two-way ANOVA followed by Tukey’s post hoc test. *P < 0.05 compared with the control group

Figure 6 shows the manifestation of seizure-like behavior (Fig. 6A) and the number of flies that were unable to recover after 10 s (Fig. 6B) in female and male flies exposed to PTZ for 24 h and subsequently submitted to the mechanical stress. PTZ increased the number of flies presenting seizure-like behavior in female (F(6, 72) = 14.72 and *p* < 0.05, Fig. 6A) and male (F(6,72) = 22.33 and *p* < 0.05, Fig. 6A) flies exposed to concentrations higher than 1 mM. Considering the number of flies without recovering after 10 s, there was a significant increase at concentrations of 1 and 10 mM in male (F(6,72) = 4.92 and *p* < 0.05, Fig. 6B). A decrease in seizure-like behavior was observed in male flies exposed to 0.1 mM PTZ (Fig. 6A).

**Fig. 7:**
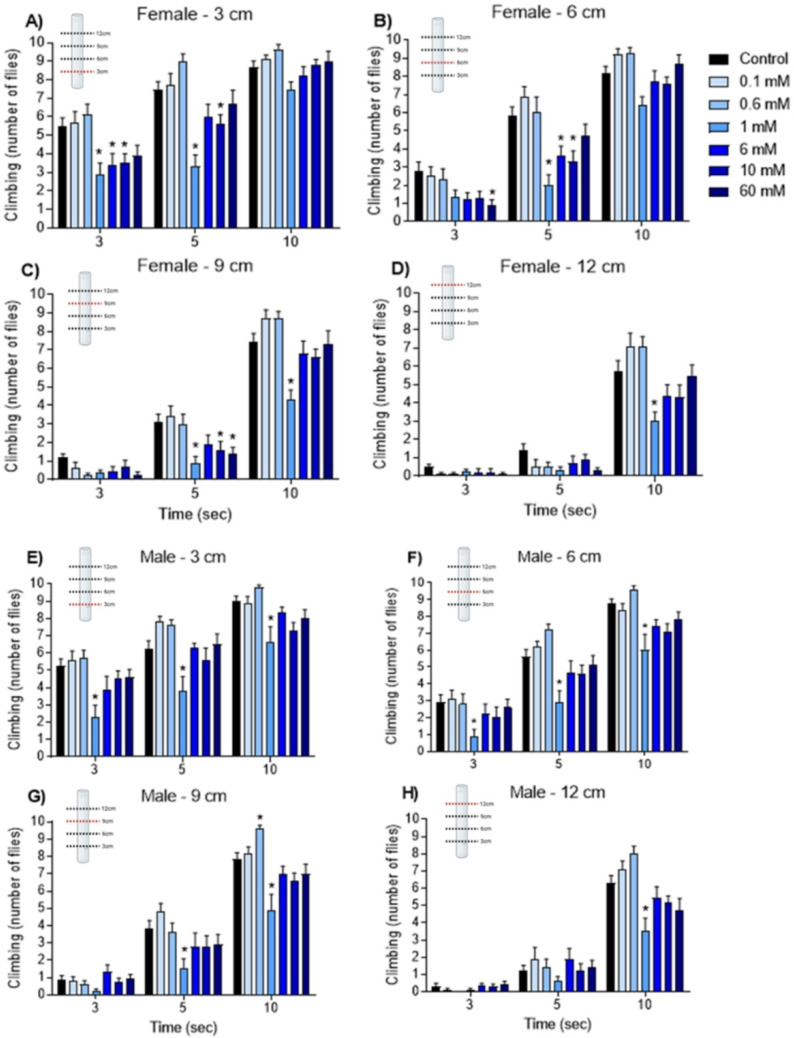
Recovery of female (**A**–**D**) and male (**E**–**H**) *Drosophila melanogaster* treated with PTZ and subjected to mechanical stress. Recovery was evaluated by the number of flies reaching the **A**, **E** 3 cm, **B**, **F** 6 cm, **C**, **G** 9 cm, and **D**, **H** 12 cm marks at 3, 5, and 10 s. Data is presented as mean ± SEM. Statistical analysis was conducted using one-way and two-way ANOVA followed by Tukey’s post hoc test. *P* < 0.05 compared with the control group at the same time point. Figures were generated using BioRender (Created inhttps://BioRender.com/h6vx781)

Regarding the ability of flies to recover locomotor activity, we standardized a method to evaluate the time necessary to reach different distances in a glass tube. In this way, the influence of PTZ on the delay to recovery after a seizure episode was evaluated. The results are depicted in Fig. 7 (A-D—females) and Fig. 7 (E–H—males). In females, PTZ affected the ability of flies to reach each distance in most of the concentrations tested, with the effect maintained at 1 mM, considering 12 cm in 10 s (F(6, 72) = 4.67 and *p*  < 0.05, Fig. 7D). In males, PTZ at a concentration of 1 mM decreased the locomotor activity of flies in the majority of analyzed times and distances, with the effect maintained at 1 mM, considering 12 cm in 10 s (F(6, 72) = 6.52 and *p*  < 0.05, Fig. 7D). Considering that the effects of PTZ at 1 mM were maintained throughout the time in both female and male flies, it was used in the subsequent analysis of sodium valproate and gabapentin effects.

### Sodium valproate decreases seizure-like behavior in male *D. melanogaster* exposed to PTZ and submitted to mechanical stress

**Fig. 8:**
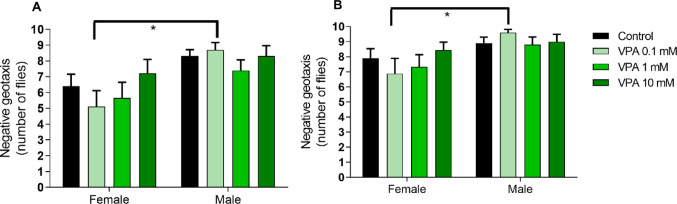
Effect of 24-h sodium valproate (VPA) exposure on the locomotor activity of *Drosophila melanogaster*. **A** Locomotor activity at 4 s and **B** at 6 s in females (n = 10) and males (n = 10). Data is presented as mean ± SEM. Statistical analysis was conducted using one-way and two-way ANOVA followed by Tukey’s post hoc test. *P < 0.05 indicates significant differences between sexes under the same treatment condition

Locomotor activity of flies exposed to sodium valproate for 24 h was evaluated by the negative geotaxis test (Fig. 8). Sodium valproate (0.1–10 mM) per se did not alter the locomotion of flies, neither when quantified at 4 s (Fig. 8A) nor 6 s (Fig. 8B). A statistically significant difference was observed between females and males at 4 s (F(1, 45) = 19.78 and *p* < 0.0001, Fig. 8A) and 6 s (F(1, 69) = 11.20 and *p* < 0.05, Fig. 8B).

**Fig. 9:**
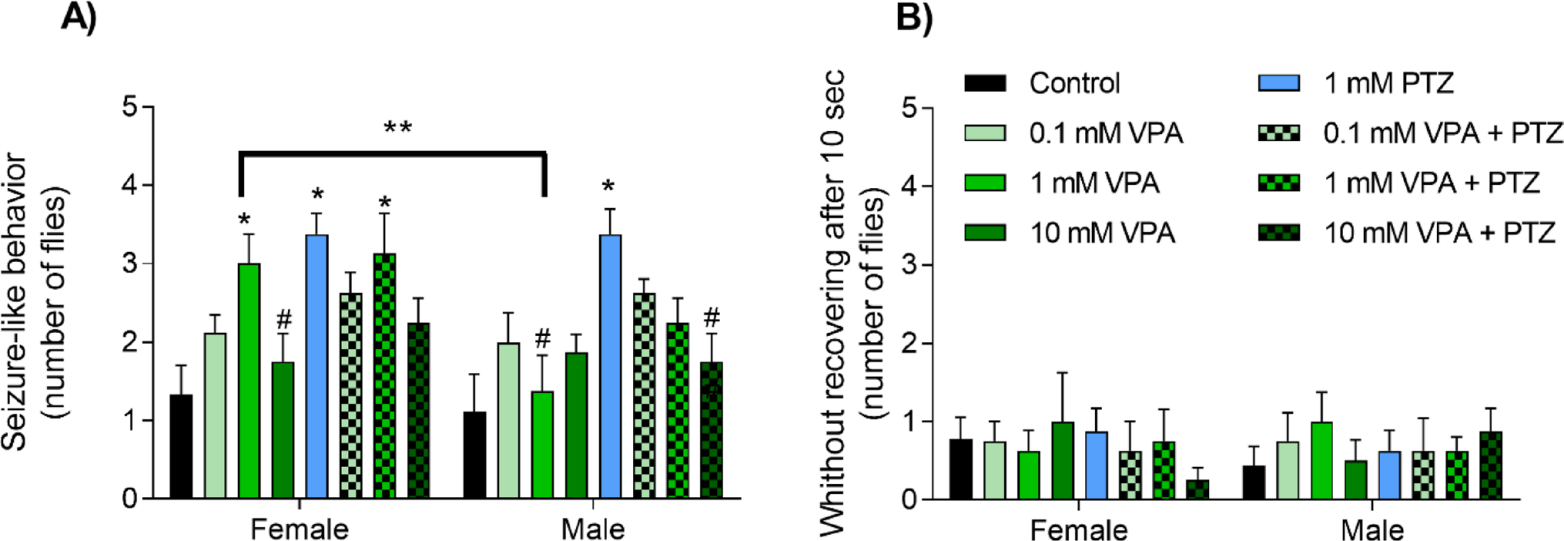
Effect of PTZ and sodium valproate (VPA) co-exposure on *Drosophila melanogaster* subjected to mechanical stress. **A** Number of flies exhibiting seizure-like behaviors. **B** Number of flies that failed to recover after 10 s. Data is presented as mean ± SEM. Statistical analysis was conducted using one-way and two-ANOVA followed by Tukey’s post hoc test. *P < 0.05 vs. control; **P < 0.05 between sexes; ^#^*P* < 0.05 vs. PTZ 1 mM group

Figure 9 shows the manifestation of seizure-like behaviors (Fig. 9A) and the number of flies that were unable to recover after 10 s (Fig. 9B) in female and male flies co-exposed with PTZ and sodium valproate for 24 h and subsequently submitted to the mechanical stress test. PTZ (1 mM) increased the number of flies presenting seizure-like behavior in female (F(7, 57) = 4.24 and * p*  < 0.05, Fig. 9A) and male (F(7, 57) = 3.98 and* p* < 0.05, Fig. 9B) flies. Sodium valproate (10 mM) reduced the effects of PTZ on seizure-like behavior only in male flies, producing an unexpected increase in this behavior in females at a concentration of 1 mM. Considering the number of flies without recovering after 10 s, no significant effects were detected (Fig. 9B).

### Gabapentin decreases seizure-like behavior in male and female *D. melanogaster* exposed to PTZ and submitted to mechanical stress

**Fig. 10:**
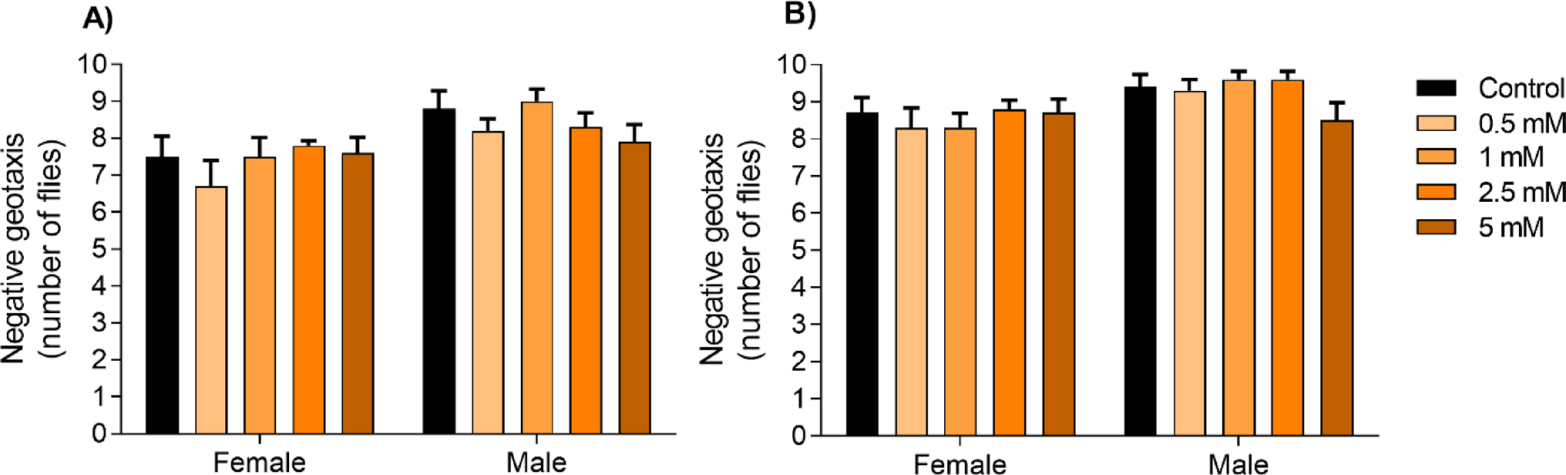
Effect of 24-h gabapentin exposure on the locomotor activity of *Drosophila melanogaster*.** A** Locomotor performance at 4 s and** B** at 6 s in females and males. Data are presented as mean ± SEM. Data were analyzed by one-way and two-way ANOVA.

The locomotor behavior of flies exposed to gabapentin for 24 h was evaluated by the negative geotaxis test (Fig. 10). No significant effects were observed, neither when quantified at 4 s (Fig. 10A) nor 6 s (Fig. 10B).

**Fig. 11:**
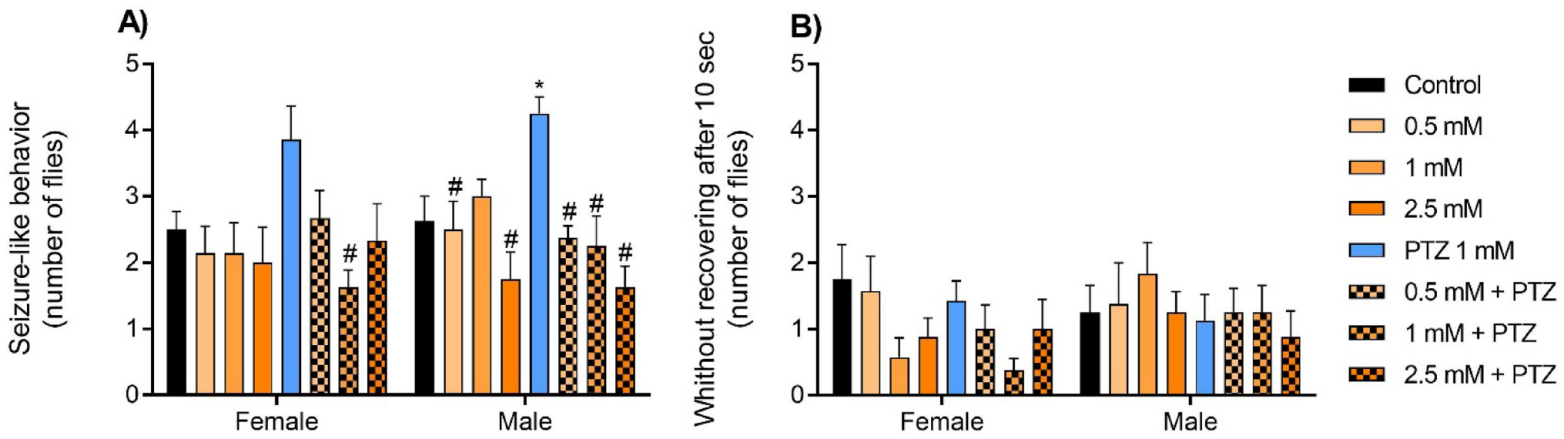
Effect of PTZ and gabapentin co-exposure on *Drosophila melanogaster* subjected to mechanical stress. **A** Number of flies exhibiting seizure-like behaviors. **B** Number of flies that failed to recover after 10 s. Data is presented as mean ± SEM. Statistical analysis was conducted using one-way and two-way ANOVA followed by Tukey’s post hoc test. *P < 0.05 vs. control; ^#^*P* < 0.05 vs. PTZ 1 mM group

Figure 11 shows the manifestation of seizure-like behaviors (Fig. 11A) and the number of flies that were unable to recover after 10 s (Fig. 11B) in female and male flies co-exposed with PTZ and gabapentin for 24 h and subsequently submitted to the mechanical tolerance test in female and male flies. PTZ (1 mM) increased the number of flies presenting seizure-like behavior in male (F(7, 54) = 5.52 and p < 0.05, Fig. 11B) flies. Gabapentin (0.5–2.5 mM) reduced the effects of PTZ on seizure-like behavior only in male flies. In females, a significant effect was detected at a concentration of 1 mM compared with the PTZ group (F(7, 49) = 2.41 and p < 0.05, Fig. 11A). Considering the number of flies without recovering after 10 s, no significant effects were detected (Fig. 11B).

## Discussion

The present study aimed to contribute to the validation of an experimental model of seizure-like behavior in *Drosophila melanogaster* for preliminary screening of anticonvulsant compounds. By combining PTZ exposure with mechanical stress, this model elicited seizure-like behaviors in both female and male flies. Seizure-like responses induced by PTZ and mechanical stress were more pronounced in males. Sodium valproate and gabapentin were effective in reducing these behaviors within this model. To our knowledge, this study demonstrates, for the first time, the effectiveness of combining chemical and mechanical stimuli to reproduce seizure-like behavior. Moreover, sodium valproate and gabapentin were effective in reducing seizure-like behavior, showing particularly high efficacy in males.

Initially, we performed the negative geotaxis test to determine whether 24-h PTZ exposure at the tested concentrations directly affected locomotor ability in flies. This step was essential to rule out the possibility that motor deficits could confound the analysis of seizure-like behaviors. No significant differences were observed in locomotor activity between female and male flies treated with PTZ. Two-way ANOVA revealed a sex effect at 4 s, with PTZ 1 mM being the main contributor to this effect. Overall, the concentration used did not impair mobility, allowing subsequent behavioral tests to focus on the seizure-like effects of PTZ. These findings are consistent with previous studies reporting no locomotor impairment in *D. melanogaster* after 48-h PTZ exposure, as assessed by both the negative geotaxis and open field tests (Soares et al. 2018).

Next, flies were pre-treated for 24 h with PTZ and subsequently subjected to two thermal or mechanical stress, to identify the most effective PTZ concentrations for inducing seizure-like behaviors. To assess the occurrence of seizure-like behaviors, the previously described phenotypic criteria were used as a standard (Parker et al. 2011). We initially tested thermal stress based on the premise that elevated temperature can trigger seizures by enhancing neuronal excitability, increasing the release of excitatory neurotransmitters, and reducing synaptic inhibition (Kim and Connors 2012). Although seizure-like behaviors were observed, no statistically significant effects were detected. In our study, the use of thermal stress showed practical limitations, including low visibility for recording behavioral phenotypes during testing.

The mechanical stress test is based on the application of intense physical stimuli capable of triggering abnormal neuronal discharges and inducing seizure-like behaviors by mimicking acute traumatic events and increasing the excitability of the nervous system (Lowenstein 2009). In this protocol, the first parameter analyzed was the occurrence of seizure-like behaviors, based on the previously described phenotype (Parker et al. 2011) followed by the quantification of flies that did not exhibit locomotor recovery within 10 s after the stimulus was applied. The results demonstrate that mechanical stress combined with PTZ induces characteristic seizure-like episodes, showing significant differences compared to the control group and similar effective concentrations in both females and males. Analysis of locomotor recovery revealed a consistent pattern between sexes. Although it was not possible to determine whether the flies exhibiting seizure-like behaviors were the same ones that failed to recover, the close correspondence in the number of affected flies suggests a possible overlap between these groups. Nevertheless, mechanical stress demonstrated reproducibility and sensitivity and was therefore adopted as the protocol for subsequent experiments.

PTZ can act as a non-competitive antagonist of GABAa receptors, which can lead to an imbalance between excitatory and inhibitory systems in the central nervous system (Akyuz et al. 2021; Monteiro et al. 2024). This promotes neuronal hyperexcitability and the induction of seizures. The responses of flies observed in the present study could be associated with findings in rodent models, where PTZ is widely used to induce myoclonic, tonic–clonic, and absence-type seizures (Löscher 2011; Jafarian et al. 2020), both in acute protocols and in kindling paradigms (Singh et al. 2021). Thus, the results observed in *D. melanogaster* reinforce the translational validity of this model.

In addition, this study employed a differential approach to assess locomotor recovery in flies following PTZ treatment and the mechanical stress test, analyzing not only the presence or absence of recovery but also the distance traveled (3, 6, 9, and 12 cm) at different time intervals (3, 5, and 10 s). This allowed a more precise and dynamic characterization of the recovery profile. Figure 7 illustrates the locomotor recovery patterns in females and males, showing that exposure to 1 mM PTZ induces alterations in both sexes. However, females exhibited more pronounced locomotor deficits at shorter distances (3 cm and 6 cm) and shorter time points (3 and 5 s). The locomotion of male flies was damaged mainly at longer distances (particularly 12 cm) and delayed time points (10 s), suggesting a differential recovery profile between female and male flies. This behavioral pattern may reflect physiological differences between sexes, with females having approximately 10% larger (Mathews et al. 2017) and a differential basal metabolic rate, resulting in distinct strategies for energy expenditure and motor performance (Videlier et al. 2021). In rodent studies, females are often characterized by greater caution (Kokras and Dalla 2014) and energy conservation, associated with a generally lower basal metabolic rate (Valle et al. 2005). Additionally, factors such as body composition and hormone influences can modulate motor performance (Oydanich et al. 2019). Therefore, it is essential to consider biological sex as a critical variable in behavioral analyses and the interpretation of experimental models. Based on the results of this set of experiments, PTZ at a concentration of 1 mM produced consistent and effective induction of seizure-like behavior in both female and male flies. Furthermore, it is known that loss of function in GABAergic neurons/signaling interferes with feeding behavior (Shaw and Webster 1979; Cheung and Scott 2017; Zhao et al. 2022), which could compromise the food intake and, consequently, the ingestion of the drugs present in the medium. As PTZ acts as a non-competitive antagonist on GABAa receptors (Monteiro et al. 2024), we avoided using higher concentrations.

The *D. melanogaster* model may be useful for screening compounds with potential anticonvulsant effects. To evaluate whether drugs clinically used for anticonvulsant effects can reduce seizure-like behavior in flies, sodium valproate and gabapentin were tested. After establishing mechanical stress and determining the most effective PTZ concentration, the effects of these two anticonvulsants, which have distinct mechanisms of action, were assessed. The first analysis focused on sodium valproate, which primarily acts by increasing GABA levels and inhibiting sodium and T-type calcium channels (Romoli et al. 2019). Initially, locomotor activity was assessed using the negative geotaxis test to rule out potential motor interference in subsequent experiments. No effects of sodium valproate were observed on the locomotor activity of female or male flies at the tested concentrations. As observed with PTZ, two-way ANOVA revealed a significant effect of sex.

Proceeding with the results related specifically to the mechanical stress test combined with 1 mM PTZ and anticonvulsants, seizure-like behavior and subsequent locomotor recovery were evaluated. A significant increase in the number of *D. melanogaster* exhibiting seizure-like behaviors was observed in the group treated with 1 mM PTZ and subjected to mechanical stress, in both females and males, compared to the control group. Treatment with sodium valproate effectively reduced the incidence of these behaviors only in males, suggesting a possible sex-dependent modulation of drug response.

Regarding the gabapentin, no locomotor alterations were observed, confirming that reductions in seizure-like behavior in subsequent tests were not due to sedation or motor deficits. Analysis of the number of flies exhibiting seizure-like behaviors and failing to recover after the mechanical stress test revealed that, in males, all gabapentin concentrations reduced the number of flies affected by PTZ and mechanical stress. In females, this effect was observed only at 1 mM of gabapentin. Regarding recovery, no significant differences were found.

Gabapentin’s mechanism of action involves blockade of the α2δ subunit of N-type calcium channels, which are associated with the release of excitatory neurotransmitters, such as glutamate (Hakami 2021b). By inhibiting these channels, gabapentin reduces neuronal excitability and calcium influx following injury (Patel and Dickenson 2016) which is particularly relevant in trauma contexts, a condition mimicked by mechanical stress in the present study. This may explain its efficacy in reducing seizure-like behaviors.

In PTZ-induced epilepsy models in rodents, mice treated with gabapentin exhibited increased latency to seizure onset, suggesting an effective protective effect of the drug on neuronal excitability (Rocha 2008). Interestingly, similar results were observed in the present study with *D. melanogaster*, where exposure to gabapentin significantly attenuated PTZ-induced seizure-like behaviors, particularly in males. This functional conservation suggests that *D. melanogaster* may serve as a useful model for the initial investigation of the pharmacological responses of potential anticonvulsant compounds.

Considering the results obtained in the present study, female flies demonstrated a higher susceptibility to seizure-like behavior and a lower response to anticonvulsants (sodium valproate and gabapentin) compared to male flies. Accordingly, sex differences in susceptibility to PTZ-induced seizures have been described in rodent models, with females exhibiting a faster onset of seizures and more severe behavioral manifestations than males when exposed to the same PTZ doses (Mejías-Aponte et al. 2002). The differences have been mainly attributed to the action of sex hormones on neuronal excitability (Scharfman and MacLusky 2006; Herzog 2008; Reddy 2013). Considering that *D. melanogaster* produces a functional analog of a steroid hormone that exerts direct effects on the nervous system (Grau and Lafont 1994; Oostra et al. 2011; Ishimoto et al. 2013), it is possible suggest that, at least in part, hormonal differences could contribute to the observed differences between female and male flies, reinforcing the translational approach of flies and rodent models.

Importantly, the inclusion of both sexes throughout the experimental design enhances the robustness and applicability of the findings, addressing a critical gap in many traditional animal models. The use of *D. melanogaster* as an experimental system for preliminary drug screening represents a promising and cost-effective alternative, particularly in the context of methods aligned with the 3Rs principles (reduction, replacement, and refinement of animal use).

In conclusion, PTZ, associated with mechanical stress, reproduces a phenotype of seizure-like behavior in *D. melanogaster*. Furthermore, this model demonstrated a response to pharmacological modulation by clinically approved drugs for epilepsy treatment, sodium valproate and gabapentin, as well as the differential responses between female and male flies. The results of the present study support the translational potential of the model for identifying promising therapeutic candidates for use in epilepsy research. Future studies investigating other compounds, and including molecular biomarkers, may further strengthen the role of this model in the initial screening and development of innovative therapies for epilepsy.

## Supplementary Information

Below is the link to the electronic supplementary material.

Supplementary Material 1

## Data Availability

The datasets generated during and/or analyzed during the current study areavailable on reasonable request.

## Abbreviations

ANOVA: Analysis of variance
PTZ: Pentylenetetrazol
SEM: Standard error of the mean
VPA: Sodium valproate
