# Supplementary material for: Sodium valproate and gabapentin reduce the seizure-like behavior induced by pentylenetetrazol and mechanical stress in Drosophila melanogaster: sex influence on behavioral responses
Source: J Neural Transm (Vienna). 2026 Apr 2;133(7):1723–37. doi: 10.1007/s00702-026-03140-0 (PMC13428723; doi:10.1007/s00702-026-03140-0)
Supplement: Supplementary file 1 — Supplementary Material 1 [file 702_2026_3140_MOESM1_ESM.docx]

**Supplementary Material 1:** Number of independent experiments (n) for each analysis. Each n corresponds to 10 flies.

| **Behavioral analysis** | **Sex** | **Concentrations (mM) and number (n) of independent experiments** |
| --- | --- | --- |
| **Negative geotaxis – PTZ** | Female | Control (n=10), 1 (n=10), 6 (n=10), 10 (n=10), 60 (n=10) |
|  | Male | Control (n=10), 1 (n=10), 6 (n=10), 10 (n=10), 60 (n=10) |
| **Thermal tolerance – PTZ** | Female | Control (n=5), 0.1 (n=5), 0.6 (n=5), 1 (n=5), 6 (n=5), 10 (n=5), 60 (n=5) |
|  | Male | Control (n=5), 0.1 (n=5), 0.6 (n=5), 1 (n=5), 6 (n=5), 10 (n=5), 60 (n=5) |
| **Mechanical resistance – PTZ** | Female | Control (n=20), 0.1 (n=10), 0.6 (n=10), 1 (n=9), 6 (n=10), 10 (n=10), 60 (n=10) |
|  | Male | Control (n=20), 0.1 (n=10), 0.6 (n=10), 1 (n=10), 6 (n=9), 10 (n=10), 60 (n=10) |
| **Negative geotaxis – VPA** | Female | Control (n=10), 0.1 (n=9), 1 (n=9), 10 (n=9) |
|  | Male | Control (n=10), 0.1 (n=10), 1 (n=10), 10 (n=10) |
| **Mechanical resistance – VPA** | Female | Control (n=9), 0.1 (n=8), 1 (n=8), 10 (n=8), 0.1+1 (n=8), 1+1 (n=8), 10+1 (n=8), PTZ 1 (n=8) |
|  | Male | Control (n=9), 0.1 (n=8), 1 (n=8), 10 (n=8), 0.1+1 (n=8), 1+1 (n=8), 10+1 (n=8), PTZ 1 (n=8) |
| **Negative geotaxis – Gabapentin** | Female | Control (n=10), 0.5 (n=10), 1 (n=10), 2.5 (n=10), 5.0 (n=10) |
|  | Male | Control (n=10), 0.5 (n=10), 1 (n=10), 2.5 (n=10), 5.0 (n=10) |
| **Mechanical resistance – Gabapentin** | Female | Control (n=8), 0.5 (n=7), 1 (n=7), 2.5 (n=8), 0.5+1 (n=6), 1+1 (n=8), 2.5+1 (n=6), 1 (n=7) |
|  | Male | Control (n=8), 0.5 (n=8), 1 (n=6), 2.5 (n=8), 0.5+1 (n=8), 1+1 (n=8), 2.5+1 (n=8), 1 (n=8) |
